# Diagnostic accuracy of intraoperative frozen section for margin evaluation of oral cavity squamous cell carcinoma

**DOI:** 10.1186/s13104-024-06698-8

**Published:** 2024-02-01

**Authors:** Javaria P. Ali, Bakhtawar Allauddin Mallick, Khushbakht Rashid, Umair Arshad Malik, Atif Ali Hashmi, Shamail Zia, Muhammad Irfan, Amir Khan, Naveen Faridi

**Affiliations:** 1 Department of Histopathology, Liaquat National Hospital and Medical College, Karachi, Pakistan; 2 Zainab Panjwani Memorial Hospital, Karachi, Pakistan; 3 Emergency Medicine, Al-Rayaz Hospital, Karachi, Pakistan; 4 Prime Cardiology of Nevada, Las Vegas, USA; 5 Department of Nephrology, Sindh Institute of Urology and Transplantation, Karachi, Pakistan; 6 Department of Internal Medicine, Aga Khan University, Karachi, Pakistan; 7 Department of Pathology, Jinnah Sindh Medical University, Karachi, Pakistan; 8 Department of Biostatistics, Liaquat National Hospital and Medical College, Karachi, Pakistan; 9 Department of Medicine, Kandahar University, Kandahar, Afghanistan

**Keywords:** Frozen section, Oral cavity squamous cell carcinoma, Diagnostic accuracy, Oral tumors, Intraoperative frozen section, Sensitivity, Specificity

## Abstract

**Objectives:**

Intraoperative frozen-section evaluation is a valuable technique for detecting positive margins intraoperatively for oral squamous cell carcinoma. We conducted this study to determine the diagnostic accuracy of frozen section in detecting margin status and the effect of tumor grade and stage on diagnostic accuracy.

**Results:**

A total of 251 biopsy-proven cases of oral squamous cell carcinoma were included in this study. The tissue specimen resected during surgery was sent to the laboratory for frozen section evaluation. The frozen section results were then compared with the permanent section results to determine the sensitivity, specificity, positive predictive value, negative predictive value, and diagnostic accuracy. The mean age of the patients included in the study was 51.65 ± 10.03 years, with male predominance (55.4%). The overall sensitivity, specificity, positive predictive value, negative predictive value, and diagnostic accuracy of frozen section were 88.81%, 94.84%, 95.20%, 88.10%, and 91.63%, respectively. We conclude that frozen section is a useful technique in determining the margin status intraoperatively in oral cancers, with high diagnostic accuracy. Moreover, certain clinical parameters such as age, gender, disease duration, and tumor stage and grade appear to affect the diagnostic accuracy of frozen section.

## Introduction

Oral cavity neoplasm is ranked as the 11th most common neoplasm in the world [1]. In Pakistan, it has been ranked as the second most common malignancy affecting both males and females [2]. The oral cavity squamous cell carcinoma (OSCC), accounts for 90% of cases of oral cavity carcinoma globally [3]. OSCC is a malignant neoplasm of the oral cavity that arises from the squamous epithelium of the oral mucosa. It shows varying degrees of squamous maturation. The major risk factors for OSCC are tobacco consumption, alcohol, betel quid, areca nut, human papilloma virus infection, and genetic predisposition [4]. Its pathophysiology is multifactorial, and major molecular alterations include TP53 and CDKN2A alterations [5]. Complete surgical resection of the tumor is the mainstay of treatment in managing OSCC, which is sufficient for early stage cancers. However, late-stage OSCC or those associated with aggressive/poor prognostic features require additional radiation or concurrent chemoradiation [6].

The anatomy of the oral cavity greatly influences the onset of dysplasia and cancer progression. From this perspective, historical research in the oral field has greatly affected our current understanding of oral cancers and clinical practice. The most commonly inflicted site in the oral cavity is the tongue, followed by the gingiva, buccal mucosa, and floor of the mouth [7].

In addition to conventional squamous cell carcinoma, there are a few variants of OSCC that are worth mentioning. Verrucous carcinoma is a low-grade variant of OSCC characterized by bulbous exoendophytic growth with pushing margins. This variant rarely metastasizes but is associated with high local recurrence. Conversely, basaloid and spindle cell squamous cell carcinomas are more aggressive. Basaloid squamous cell carcinoma is characterized by basaloid palisading cells with central necrosis, whereas spindle cell squamous cell carcinoma is distinguished by sheets of spindle cells resembling sarcoma. Finally, adenosquamous and adenoid squamous cell carcinomas are other rare variants of squamous cell carcinoma. The latter has an adenoid pattern and generally occurs on sun-exposed skin of the head and neck, but rarely occurs in the oral cavity [8].

Certain biological and morphological prognostic factors, such as tumor grade, tumor stage, depth of invasion, perineural and perivascular invasion, extracapsular spread, and resected margins, determine the overall outcome of the disease. There are three grades (depending upon the degree of differentiation and four stages of OSCC. These stages depend upon tumor size, depth of invasion, nodal metastasis, skin and bone involvement. Very advanced local disease is considered when tumor involves masticator space, pterygoid plates, skull base, or encase the internal carotid artery [9]. Among all these prognostic parameters, the resected margin status is an independent prognostic factor in determining disease-specific survival [10]. In surgical oncology, complete resection of the primary tumor with a negative tumor margin is a prime prognostic factor because the presence of any residual neoplastic tissue results in positive surgical margins and is associated with local recurrence and poor prognosis [11]. It has been reported that tumors of the oral cavity have a higher probability of positive surgical margins [12]. Margin-negative primary radical resection of OSCC is of utmost importance for preventing recurrence [13]. Moreover, if the margin is positive, a second surgery to remove the residual tumor is difficult because of inflammation, granulation tissue, and fibrosis. Intraoperative frozen section (FS) evaluation is an important technique and is highly accurate in providing a quick pathological assessment of tumor margin intraoperatively, as it allows the determination of positive margin and its adequate resection before surgical closure [14]. The achievement of adequate resection margin in radical resection of cancers of oral cavity has been critical; hence, FS evaluation has been widely accepted in oral cavity cancer surgeries to obtain adequate resection of the tumor cell with negative margins [15, 16].

Several studies have reported that FS evaluation has a high accuracy rate [17, 18]. However, it is unclear whether the stage and grade of the tumor affect the diagnostic accuracy of FS for detecting margin status in OSCC. Therefore, we conducted this study to determine the diagnostic accuracy of FS in determining the margin status of OSCC with respect to tumor grade and stage.

## Methods

This was a retrospective, cross-sectional study conducted on 251 biopsy-proven cases of OSCC of OC. The study was conducted at the Department of Histopathology, Liaquat National Hospital, Karachi, Pakistan from November 2021 to April 2022. Clinicopathological data of the cases reported at our institute during this period were retrieved from the institute archive. All biopsy-proven cases of OSCC were enrolled in the study, and all of these patients were included. All patients underwent clinical examination and workup, including computed tomography (CT) scan and incisional biopsy, and then underwent surgical resection of the primary tumor at our hospital. Cases with missing clinical and surgical data were excluded from the study. Moreover, patients who received neoadjuvant chemotherapy or radiotherapy before surgical resection were also excluded from the study. Patients with multiple primary tumors and residual diseases were excluded from the study. After resection of the neoplastic tissue, the margins of the samples were labeled and sent to the histopathology laboratory.

In the laboratory, the samples were grossly examined for color, texture, consistency, and suture to mark the anatomical position. The tissue samples were placed in a cryostat machine with a set temperature of -22 °C for freezing; the frozen samples were then thinly sliced into 5–6 micron diameter sections via a microtome. The sliced tissue samples were fixated in 95% ethanol, followed by affixing the tissue sample on glass slides, which were subsequently stained with hematoxylin and eosin and were interpreted by a senior pathologist. The results were communicated to the surgeon as positive and negative by phone within 20 min. In case of a positive margin on FS, further resection was performed. The remaining tissue after FS and any additional tissue resected during surgery were fixed and embedded in paraffin wax to prepare formalin-fixed-paraffin-embedded blocks, which were sliced, stained with hematoxylin and eosin, and studied under a microscope by a senior pathologist. The results of FS and permanent section were compared to determine the sensitivity and specificity of FS in detecting the margin status.

### Data analysis

IBM Statistical Package for the Social Sciences (SPSS) Version 26.0 was used for data analysis. Sensitivity, specificity, positive predictive value (PPV), negative predictive value (NPV), and to calculate the diagnostic accuracy for FS diagnosis by 2 × 2 tables the final (paraffin) section diagnosis was used as the gold standard. The mean and standard deviation for the patient’s age and disease duration were calculated.

## Results

A total of 251 cases of OSCC were included in the study. Table 1 illustrates the descriptive statistics of the study population. Our data demonstrated that the mean age of the patients at the time of diagnosis were 51.65 51.65 ± 10.03 years, with the majority (45.4%) of patients being in the age group of 51–60 years. The disease was found to be more prevalent among males, accounting for 55.4% of cases. The mean duration of the disease was 4.35 ± 1.01 months. The study demonstrated that in most of the cases, approximately 49% were found to have stage II disease, followed by stage I disease, which was diagnosed in 35% of cases. The most common tumor grade in our study was grade 2n 56.2% of cases, followed by grade 1 (28.3%). In approximately 49.8% of the cases, the FS margins were found to be positive for metastatic cancer, whereas 53.4% were positive for metastatic carcinoma on final (paraffin) histology.

**Table 1:** Descriptive statistics of the study population (*n* = 251)

Clinicopathological features	Mean ± SD/n(%)
**Gender**	
Male	139(55.4)
Female	112(44.6)
**Age (years)**	
Mean ± SD	51.65 ± 10.03
**Age groups**	
≤ 40 years	37(14.7)
41–50 years	68(27.1)
51–60 years	114(45.4)
> 60 years	32(12.8)
**Disease duration (months)**	
Mean ± SD	4.35 ± 1.01
**Disease duration, group**	
≤ 4 months	122 (48.6)
> 4 months	129(51.4)
**Tumor stage**	
Stage-I	88(35.0)
Stage-II	123(49)
Stage-III	24(9.6)
Stage-IV	16(6.4)
**Tumor grade**	
Grade 1	71(28.3)
Grade 2	141(56.2)
Grade 3	39(15.5)
**Frozen section margin status**	
Positive	125(49.8)
Negative	126(50.2)
**Final (paraffin) margin status**	
Positive	134(53.4)
Negative	117(46.6)

SD: standard deviation

Table 2 illustrates the sensitivity, specificity, PPV, NPV, and diagnostic accuracy of FS in detecting margin positivity using paraffin sections as the gold standard. The overall sensitivity, specificity, PPV, and diagnostic accuracy of intraoperative FS were 88.81%, 94.84%, 95.20%, 88.10%, and 91.63%, respectively.

**Table 2:** Comparison of frozen section with final (paraffin) for the evaluation of margins of oral squamous cell carcinoma

Variables	Frozen section margin status	Final (paraffin) section margin status		Sensitivity	Specificity	PPV	NPV	Diagnostic accuracy
		Positive	Negative					
Overall	Positive	119	6	88.81%	94.84%	95.20%	88.10%	91.63%
	Negative	15	111					

PPV: positive predictive value; NPV: negative predictive value

Table 3 illustrates the sensitivity, specificity, PPV, NPV, and diagnostic accuracy of FS with respect to clinical parameters such as gender, age, and disease duration. The sensitivity, specificity, PPV, NPV, and diagnostic accuracy of FS for males were 90.67%, 95.31%, 95.77%, 89.71%, and 92.81%, respectively, whereas for females it was 86.44%, 94.34%, 94.44%, 86.21%, and 90.18%, respectively. With respective to age ≤ 50 years it was found to be 94.64%, 93.75%, 94.64%, 93.75%, and 94.23%, respectively, whereas for age > 50 years, it was found to be 84.62%, 95.65%, 95.65%, 84.62%, and 89.80%, respectively. With respect to disease duration of ≤ 4 months the sensitivity, specificity, PPV, NPV, and diagnostic accuracy of FS were found to be 82.43%, 95.83%, 96.83%, 77.97%, and 87.70%, respectively, whereas for disease duration > 4 months it was found to be 96.67%, 94.20%, 93.55%, 97.01%, and 95.35%, respectively.

**Table 3:** Comparison of frozen section with final (paraffin) for the evaluation of margins of oral squamous cell carcinoma with respect to clinical parameters

Variables		Frozen section margin status	Final (paraffin) section margin status		Sensitivity	Specificity	PPV	NPV	Diagnostic accuracy
			Positive	Negative					
Gender	Male	Positive	68	3	90.67%	95.31%	95.77%	89.71%	92.81%
		Negative	7	61					
	Female	Positive	51	3	86.44%	94.34%	94.44%	86.21%	90.18%
		Negative	8	50					
Age	≤ 50 years	Positive	53	3	94.64%	93.75%	94.64%	93.75%	94.23%
		Negative	3	45					
	> 50 years	Positive	66	3	84.62%	95.65%	95.65%	84.62%	89.80%
		Negative	12	66					
Disease Duration	≤ 4 months	Positive	61	2	82.43%	95.83%	96.83%	77.97%	87.70%
		Negative	13	46					
	> 4 months	Positive	58	4	96.67%	94.20%	93.55%	97.01%	95.35%
		Negative	2	65					

PPV: positive predictive value; NPV: negative predictive value

Table 4 illustrates the sensitivity, specificity, PPV, NPV, and diagnostic accuracy of FS with respect to tumor stage and grade. The sensitivity of FS for tumor stages I, II, and III/IV was 78.38%, 89.70%, and 100%, respectively, indicating that the sensitivity of FS increased with tumor stage. Similar findings were noted for specificity, PPV, and NPV. Similarly, the diagnostic accuracy of FS for stage I tumors was 84.09%, whereas it was 94.30% and 100% for stage II and stage III/IV, respectively, indicating that the diagnostic accuracy increases with the advanced tumor stage. The sensitivity of FS with respect to grade 1, grade 2, and grade 3 tumors was 0.00%, 87.20%, and 94.80%, respectively. Similarly, the diagnostic accuracy of intraoperative FS for grade 1 tumors was 90.10%, whereas it was 91.50% and 94.80% for grade 2 and grade 3 tumors, respectively. Thus, demonstrating that similar to the tumor stage, the sensitivity and diagnostic accuracy of FS improved as the tumor grade increased.

**Table 4:** Comparison of frozen section with final (paraffin) for the evaluation of margins of oral squamous cell carcinoma with respect to tumor grade and stage

Variables		Frozen section margin status	Final (paraffin) section margin status		Sensitivity	Specificity	PPV	NPV	Diagnostic Accuracy
			Positive	Negative					
Tumor stage	Stage-I	Positive	29	6	78.38%	88.24%	82.86%	84.91%	84.09%
		Negative	8	45					
	Stage-II	Positive	61	0	89.70%	100%	100%	88.70%	94.30%
		Negative	7	55					
	Stage-III/IV	Positive	29	0	100%	100%	100%	100%	100%
		Negative	0	11					
Tumor grade	Grade 1	Positive	0	6	0.00%	91.40%	0.00%	98.50%	90.10%
		Negative	1	64					
	Grade 2	Positive	82	0	87.20%	100%	100%	79.60%	91.50%
		Negative	12	47					
	Grade 3	Positive	37	0	94.80%	0.00%	100%	0.00%	94.80%
		Negative	2	0					

PPV: positive predictive value; NPV: negative predictive value

## Discussion

This study was conducted to evaluate the diagnostic accuracy of FS evaluation in determining margin status in OCSCC. We concluded that the overall diagnostic accuracy of FS in determining the margin status in OCSCC is effective with a sensitivity, specificity, PPV, NPV, and diagnostic accuracy of 88.81%, 94.84%, 95.20%, 88.10%, and 91.63%, respectively.

The primary aim of head and neck tumor surgery is complete tumor resection with a negative margin, which, however, can be missed by surgeons because of the presence of microscopic disease invisible to the naked eye. A positive margin of 5 mm is associated with a higher probability of local recurrence [19]. To reduce the recurrence rate, adequate margin clearance is essential in head and neck tumors, and intraoperative FS is widely used by surgeons to achieve clear margins [20]. Several studies have previously proposed that intraoperative FS evaluation is an important and high-yield technique in ensuring adequate clearance of the tumor in OSCC [18, 21]. Previous studies estimated the accuracy rate of FS of the head and neck to range between 96% and 98% [15, 22].. In addition, the sensitivity of FS in assessing margins in OSCC in our study was found to be slightly lower (91.63%) than the range proposed by previous studies.

A study conducted by Asoda et al. [23] evaluated the clinical value of entire-circumferential intraoperative FS for the complete resection of SCC of the tongue in 276 patients. He concluded that FS is a valuable technique for achieving complete resection of tumors with negative margins with 100% sensitivity and 100% NPV. Moreover, he concluded that FS, when performed with iodine staining, provided added diagnostic accuracy. Similar to our study, a study by Wasif et al. [19] on 94 patients with OSCC, found the sensitivity of FS to be 50% whereas the specificity was calculated to be 99.8%, PPV and NPV were found to be 75% and 99.3%, respectively, and the overall diagnostic accuracy was found to be 99%, concluding that FS is a very useful tool that can help surgeons intraoperatively whether adequate clear surgical margins have been obtained or not.

Other studies conducted previously have negated the role of FS in the intraoperative determination of the surgical margin [11]. There is contradicting evidence on the association of FS with improved overall prognosis, with a few researchers finding no association between FS and overall outcome, whereas a few found prognostic benefits associated with FS [24]. Similar to our study, a previous study found that the sensitivity of intraoperative FS histology compared with final specimen histology was high [25]. Algadi et al. [11] conducted a study for comparison, toluidine blue (TB) versus frozen section for assessment of mucosal tumor margins in OSCC, they concluded that FS had a sensitivity of 50.0%, specificity of 100.0%, PPV of 100.0%, NPV of 97.8%, and accuracy of 97.9%. In contrast to our study, they found FS to be a moderately useful technique for intraoperatively assessing tumor margins. They proposed that TB is a more sensitive technique and that screening of margins with TB before FS sampling may improve the identification of more tumor margins. Another study by Mair et al. [26] also reported a low FS sensitivity of 45.4% in assessing margin status.

### Limitations

Our study had a few limitations. Because the study was conducted and included data from a single institute, the sample size was limited. Moreover, this was a retrospective study; therefore, follow-up of the patients was not performed to determine the disease-free survival rate. The risk factors for OSCC were also not evaluated.

## Conclusion

Intraoperative FS is a valuable technique for determining the margin status in OSCC, with high sensitivity, specificity, PPV, NPV, and diagnostic accuracy. Moreover, a positive association was found between tumor grade and tumor stage with the sensitivity and diagnostic accuracy of FS, i.e., the sensitivity and accuracy increased with tumor grade and stage. Moreover, certain clinical parameters such as age, gender, and disease duration also seem to slightly influence the sensitivity and accuracy of FS.

Furthermore, we recommend multicenter prospective studies to accurately determine the diagnostic accuracy of FS in OSCC. Moreover, guidelines should be developed to implement FS in head and neck surgeries and how to accurately evaluate FS margins, for instance, the number of sections to be examined. Furthermore, the role of FS in assessing sentinel node metastasis should also be evaluated to prevent radial neck dissection.

## Data Availability

No datasets were generated or analysed during the current study.

## Abbreviations

OSCC: Oral Squamous cell carcinoma
FS: Frozen section
CT: Computed tomography
PPV: Positive predictive value
NPV: Negative predictive value
